# Analysis of 16S rRNA genes reveals reduced Fusobacterial community diversity when translocating from saliva to GI sites

**DOI:** 10.1080/19490976.2020.1814120

**Published:** 2020-10-15

**Authors:** Miles Richardson, Jihui Ren, Mara Roxana Rubinstein, Jamila A. Taylor, Richard A. Friedman, Bo Shen, Yiping W. Han

**Affiliations:** a Department of Systems Biology, Columbia University Irving Medical Center, New York, NY, USA; b Division of Periodontics, College of Dental Medicine, Columbia University Irving Medical Center, New York, NY, USA; c Department of Biomedical Informatics, Vagelos College of Physicians & Surgeons, Columbia University Irving Medical Center, New York, NY, USA; d Biomedical Informatics Shared Resource, Herbert Irving Comprehensive Cancer Center, Vagelos Columbia University Irving Medical Center, New York, NY, USA; e Department of Gastroenterology and Hepatology, Cleveland Clinic, Cleveland, OH, USA; f Department of Microbiology, Vagelos College of Physicians & Surgeons, Columbia University Irving Medical Center, New York, NY, USA; g Department of Microbiology, Columbia University Irving Medical Center, New York, NY, USA; h Herbert Irving Comprehensive Medical Center, Columbia University Irving Medical Center, New York, NY, USA; i Institute of Human Nutrition, Columbia University Irving Medicine Center, New York, NY, USA

**Keywords:** *Fusobacterium*, 16S rRNA, saliva, gastric aspirate, colon aspirate, ileal pouch aspirate, GI tract, inflammatory bowel disease

## Abstract

*Fusobacterium nucleatum* is a Gram-negative oral commensal anaerobe which has been increasingly implicated in various gastrointestinal (GI) disorders, including inflammatory bowel disease, appendicitis, GI cancers. The oral cavity harbors a diverse group of *Fusobacterium*, and it is postulated that *F. nucleatum* in the GI tract originate from the mouth. It is not known, however, if all oral *Fusobacterium* translocate to the GI sites with equal efficiencies. Therefore, we amplified 16S rRNA genes of *F. nucleatum* and *F. periodonticum*, two closely related oral species from matched saliva, gastric aspirates, and colon or ileal pouch aspirates of three patients with inflammatory bowel disease (IBD) and three healthy controls, and saliva alone from seven patients with either active IBD or IBD in remission. The 16S rRNA gene amplicons were cloned, and the DNA sequences determined by Sanger sequencing. The results demonstrate that fusobacterial community composition differs more significantly between the oral and GI sites than between different individuals. The oral communities demonstrate the highest level of variation and have the richest pool of unique sequences, with certain nodes/strains enriched in the GI tract and others diminished during translocation. The gastric and colon/pouch communities exhibit reduced diversity and are more closely related, possibly due to selective pressure in the GI tract. This study elucidates selective translocation of oral fusobacteria to the GI tract. Identification of specific transmissible clones will facilitate risk assessment for developing *Fusobacterium*-implicated GI disorders.

## Introduction

Genus *Fusobacterium* are Gram-negative obligate anaerobic bacilli with tapered or fusiform ends and produce butyrate as a metabolic end product. There are currently 13 species, among which *Fusobacterium nucleatum* and *Fusobacterium periodonticum* are two closely related species that normally dwell in the oral cavity.^1^ Under diseased conditions, however, they can translocate to extra-oral sites causing infection and inflammation.^2^ *F. nucleatum* is one of the most prevalent species isolated from human infections, having been implicated in atherosclerosis, adverse pregnancy outcomes, rheumatoid arthritis, and organ abscesses and infections.^3^ In recent years, *F. nucleatum* has been increasingly associated with GI disorders, including inflammatory bowel disease (IBD), appendicitis, and esophageal, pancreatic, and colorectal cancers (CRC).^3–13^ The presence of *F. nucleatum* in cancers is often associated with worse forms and poor prognosis.^4,13–15^ *F. nucleatum* colonizes and invades CRC cells and stimulates cancer growth through binding of its unique FadA adhesin to E-cadherin.^16^ It modulates the tumor micro-environment, confers chemoresistance, and promotes CRC metastasis.^17–21^ *F. nucleatum* exacerbates CRC progression via a positive feedback loop between FadA and Annexin A1, which then activates Wnt/β-catenin signaling.^22^ Although *F. nucleatum* can disseminate through hematogenous transmission,^23,24^ studies have demonstrated enrichment of *F. nucleatum* and *fadA* gene in the fecal microbiome of CRC patients compared to the normal controls, suggesting translocation through the GI tract.^20,25,26^

*F. nucleatum* is a highly diverse species, consisting of five subspecies: *animalis, fusiforme, nucleatum, polymorphum*, and *vincentii*.^1^ Each individual may harbor multiple strains of different subspecies in the oral cavity. It was reported that up to seven different genotypes could be detected in the same oral cavity and up to four different genotypes were observed within a single site.^27^ Previous report showed that *F. nucleatum* detected in intrauterine infection predominantly belongs to subspecies *animalis*, followed by subsp *polymorphum*.^2^ *F. nucleatum* subsp *animalis, polymorphum, nucleatum, and vincentii* have all been detected in CRC.^28,29^ When matched saliva and CRC samples were analyzed, more *Fusobacterium* strains were detected in saliva than in CRC.^28^ These observations suggest that not all *Fusobacterium* disseminate to extra-oral sites with equal efficiencies.

16S rRNA is often used to identify species. Microorganisms with >97% sequence identity of 16S rRNA gene are considered the same species.^30^ However, even within species of closer than 97% similarity, there can be significant variations, which may account for differences in host specificity^31^ and ecological niche.^32^ There is considerable genomic variability among *F. nucleatum*, with the average nucleotide identity (ANI) between subspecies of less than 93%.^33^ Thus, it is important to not only distinguish between subspecies, but also identify relevant strains within the subspecies.

In this study, we examine the abundance patterns of different *Fusobacterium* in matching samples of saliva and GI (gastric, colon, and ileal pouch) aspirates from IBD patients and healthy controls. We accomplish this by using Minimum Entropy Decomposition (MED) to assemble representative sequences of Sanger-sequenced 16S rRNA genes. We reveal that *Fusobacterium* translocate through the GI tract in distinct sub-communities. Investigation of transmissible strains will help identify unique virulence factors and individuals at risk for developing GI disorders.

## Materials and methods

### Sample collection

Matching samples of saliva, gastric aspirate, and colon or ileal pouch aspirate were collected from three IBD patients and three healthy controls at the endoscopy suite at Cleveland Clinic in Cleveland, Ohio (Table 1). Saliva was collected prior to the endoscopy procedures by having the patients spit into sterile collection vials. Gastric aspirates were collected during endoscopy, and colon or ileal pouch aspirates were collected during colonoscopy, respectively. The ileal pouch is made from a loop of distal ileum that serves as a fecal reservoir in patients with colectomy (i.e. removal of colon) resulting from ulcerative colitis. The patients underwent polyethylene glycol-based colonic preparation prior to colonoscopy. Saliva alone was also collected from seven additional IBD patients (Table 1). All samples were stored at −80°C until use. This study was approved by the Internal Review Board at Cleveland Clinic (IRB 06–673). Written informed consent was obtained prior to patient enrollment and sample collection. The specimens were de-identified before being analyzed at Columbia University.

**Table 1.** Participants and specimen description

Subject No.	Samples collected^a^	Disease characteristics^b^	Group
1	saliva, gastric aspirate, colon aspirate	Healthy participant	Healthy
2	saliva, gastric aspirate, colon aspirate	Healthy participant	Healthy
3	saliva, gastric aspirate, colon aspirate	Healthy participant	Healthy
4	saliva, gastric aspirate, pouch aspirate	Normal stomach/pouchitis	IBD
5	saliva, gastric aspirate, pouch aspirate	Normal stomach/CD of pouch	IBD
6	saliva, gastric aspirate, pouch aspirate	CD of stomach and colon	IBD
2667	saliva	Normal pouch	IBD^*^
2704	saliva	Normal pouch	IBD^*^
2714	saliva	Normal pouch	IBD^*^
2674	saliva	Pouchitis	IBD
2678	saliva	CD of pouch	IBD
2705	saliva	CD of pouch	IBD
2706	saliva	CD of pouch	IBD

^a^Saliva was collected by patients spitting into collection vials in the endoscopy suite prior to the endoscopy procedures. Gastric and colon or ileal pouch aspirates were collected during the endoscopy and colonoscopy procedures, respectively.

^b^Ileal pouch is made from a loop of distal ileum that serves as a fecal reservoir in patients with colectomy (i.e. removal of colon) resulting from ulcerative colitis. Pouchitis is the nonspecific inflammation of the ileum reservoir with bacterial etiology. Due to the pouch surgery, some patients with a preoperative diagnosis of ulcerative colitis may develop de novo Crohn’s disease (CD) of the pouch. In contrast to bacterial etiology of pouchitis, CD of the pouch is believed to result from a combination of factors of autoimmune, surgical ischemia, and microbiome. Pouchitis along with CD and UC are all considered as different forms of IBD.

^*^In remission.

### DNA extraction, PCR, cloning, and DNA sequencing

DNA from saliva and gastric aspirates was extracted by the phenol/chloroform/isoamyl alcohol method.^34^ Briefly, the samples were centrifuged, and the pellets were collected and dissolved in lysis buffer (10 mM Tris HCl pH8.0, 1 mM EDTA, 1% SDS). After adding one volume of phenol:chloroform:isoamyl alcohol (25:24:1), the samples were transferred to tubes containing glass-beads (Microbead tubes, MO BIO, Carlsbad, CA, USA) and beaten vigorously for 2 min and repeat 5 times. Following centrifugation, the upper aqueous phase was collected, mixed with 0.5 volume of 7.5 M NH_4_OAc and -2.5 volume of 100% ethanol, and stored at –20°C overnight before centrifugation to precipitate DNA. The DNA pellets were washed with 70% ethanol and resuspended in TE buffer. DNA was cleaned further using Genomic DNA Clean and Concentrator Kit (ZYMO Research, Irvine, CA, USA) following the manufacturer’s instructions. QIAamp Fast DNA Stool Mini Kit (Qiagen, Hilden, Germany) was used to extract DNA from colon/pouch aspirates because it can effectively remove PCR inhibitors that are often abundant in these samples. Microbial communities obtained from these two methods cluster closely in the dendrogram graph, thus are similar.^35^ *Fusobacterium* 16S rRNA gene was amplified by PCR using forward primer 785 F (5ʹGGATTAGATACCCTGGTAGTC3ʹ) and backward primer BWR1 (5ʹCTCTTTCGTATTAAGACTCCA3ʹ), which specifically amplify the 16S rRNA genes of both *F. nucleatum* and *F. periodonticum*, two most closely related oral species,^36^ generating an amplicon containing the second half of the 16S rRNA gene, starting near position 785, and including part of the internal transcribed spacer (ITS) region downstream of the 16S rRNA gene. PCR amplicons were cloned into plasmid pCR2.1-TOPO (Invitrogen) and transformed into competent *E.coli* (One Shot TOP10, Invitrogen, Carlsbad, CA, USA). The bacteria were plated onto LB plates containing 50 µg/ml ampicillin and 40 µg/ml 5-bromo-4-chloro-3-indolyl-beta-D-galactopyranoside (X-gal). The plasmids were purified using Purelink Quick Plasmid DNA Miniprep Kit (Invitrogen), followed by Sanger sequencing using M13F and M13R primers. The number of clones sequenced from each sample is listed in Supplementary Table 1.

### Quality filtering and sequence alignment

The Sanger sequencing data were converted to fastq files using biopython,^37^ and quality filtered in the following manner. Forward and reverse sequences were trimmed to 600 bases long, as visual examination confirmed that this region has high accuracy, with Phred > 30. Additionally, 70 bases were removed from the ends to eliminate adapter errors. Quality summaries for each patient are presented in Supplementary Figure 1. Paired ends were then merged using vsearch.^38^ All sequences were aligned to the second-half of *Fusobacterium* 16S rRNA and a portion of the internal transcribed spacer (ITS) region downstream of the 16S rRNA gene using PyNAST,^39 ^This merged sequence was then trimmed to remove the ITS region due to its high variability, producing a product of 792 bp. The sequence counts from each sample are presented in Supplementary Table 1. A total of 383 sequences remained after quality filtering. These sequences are deposited in Genbank under the reference number SUB7794064, with MT780937 – MT781319 as accession numbers for each individual sequences.

### Minimum entropy decomposition (MED)

The 792 bp fragments were used for MED and tree generation. In order to identify low-level species or strains, the aligned sequences were clustered by MED,^40^ an automated version of oligotyping.^41^ This method searches for nucleotide positions in the input sequence that have high divergence, and iteratively decomposes them into groups of sequences, called nodes, which are representative sequences used for BLAST search. MED was run with parameters of minimum substantive abundance (-M) of 3 and relocation of outliers, with all other parameters set to their defaults. One sequence was removed due to too low a substantive abundance (1), and another was removed because of excess variations in nucleotide sites. A total of 381 sequences remained for analysis (Supplementary Table 1).

### Tree generation

Reference fusobacterial sequences from NCBI GenBank^42^ were chosen by acquiring the top two BLAST hits for each node. Many of the top two sequences were not unique and matched multiple nodes. These reference sequences were added to the aligned sequences, and phylogenetic relationships were determined using FastTree.^43^ The tree was plotted using ggtree,^44^ using reference sequences obtained from BLAST for subspecies identities. Node colorations were assigned manually based on monophyletic relationships in the tree.

### Ecological and statistical analyses

The difference between communities from different sites and/or subjects was quantified by the Bray–Curtis dissimilarity^45^ and their statistical significance estimated using ANOSIM.^46^ Rarefaction curves with the Chao1 richness as the dependent variable were used to compare species richness between sites.^47,48^ This was accomplished by pooling reads according to sample sites, sampling reads without replacement from each site pools one at a time, and then calculating the Chao1 diversity using the *estimate_richness* function in phyloseq.^49^ Mann–Whitney tests were performed using the Wilcox test function in base R. Kruskal–Wallis tests were performed with the *kruskal.test* function. ANOSIM was run using the *anosim* function from vegan.^50^ Heatmaps, rarefaction curves, and PCoA plots were made using ggplot2,^51^ all of which were performed using R 3.3.4. Unless otherwise noted, all analysis was performed using the matched saliva, gastric aspirate, and colon or ileal aspirate samples (sample triads) from the six individuals with complete sampling.

### Enrichment analysis

Enrichment of a certain node at a body site was performed using the binomial test, using the *binom.test* function.^52^ In brief, for each node (i.e. strain), the site with the greatest abundance was compared pairwise to the other two body sites, using a one sided (greater) binomial test with *p* = 1/3, the number of trials (N) equal to the abundance of the node. If it was significantly greater relative to both of the other two body sites, it was marked as significantly enriched in that body site.

## Results

### Long-read sequences for assessing diversity of Fusobacterium

Fusobacteria-specific PCR primers, combined with MED^40^ allowed for fine-scale interrogation of fusobacterial diversity. MED is an automated version of oligotyping^41^ that uses single nucleotide differences in the 16S rRNA gene to group sequences into sub-groups called oligotypes. The group partitioning was aided by the ability to utilize a 792 bp amplicon of the 16S rRNA gene. This is due to the observation that fusobacterial 16S rRNA has sites of high entropy along the entire amplicon (Supplementary Figure 2), which cannot be resolved by only assessing a 250 bp fragment, as is typical in Illumina paired-end sequencing.^53^ More sites of high entropy allow for increased power in discerning sequences, allowing for the recovery of unique fusobacterial sequences, or nodes. After quality control and performing MED, 381 sequences formed 28 unique nodes, each representing a unique strain of fusobacteria.

### Evaluation of the completeness of sampling and alpha diversity

**Figure 1.**
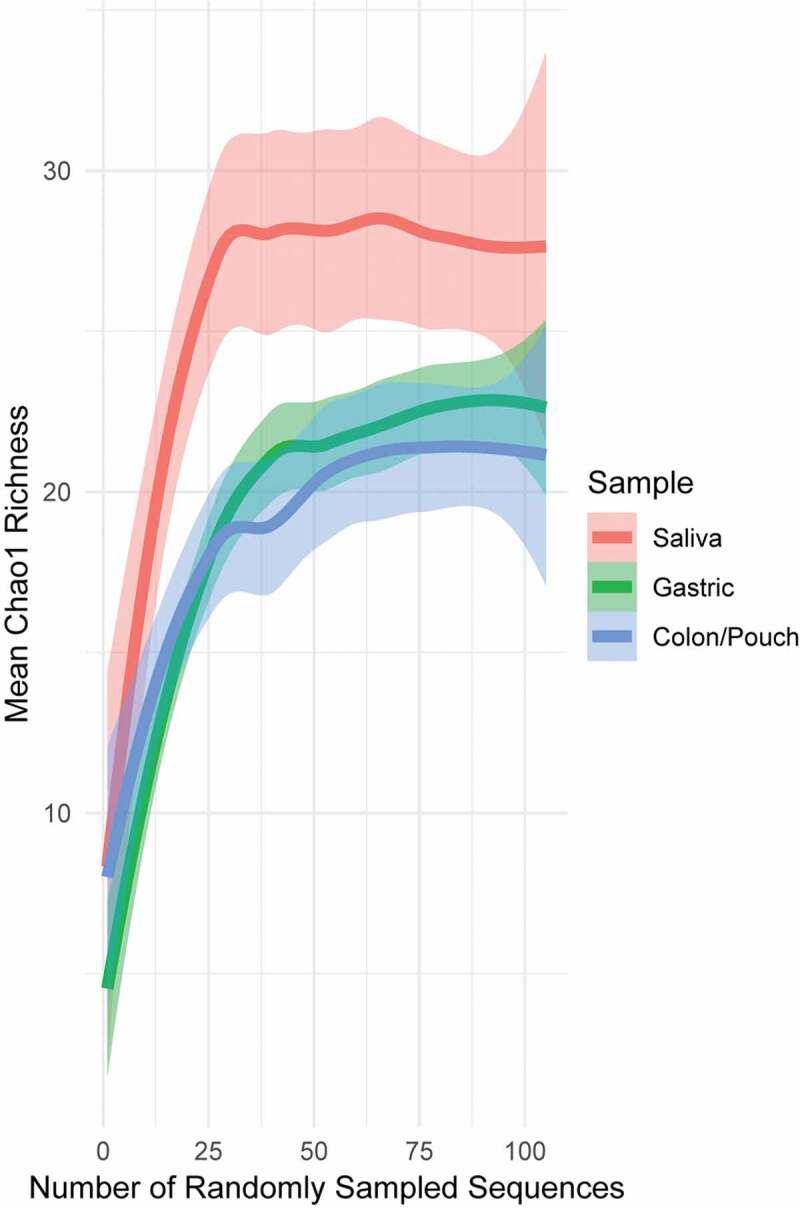
Rarefaction curves generated from pooled sequences from 3 IBD patients and 3 healthy controls with matching sample triads, i.e. saliva, gastric aspirate, colon/pouch aspirate. Lines are drawn by LOESS (locally estimated scatterplot smoothing), with lighter colored areas surrounding the lines as the standard error intervals. Rarefaction curves averaged across individual samples can be seen in Supplementary Figure 3. The pool of saliva samples is more diverse compared to gastric and colon/pouch samples

In all sequencing and all sampling-based methods, it is not unusual to have missing members of the microbial community due to limitations of sampling. This can result in a misleading presentation of diversity, as part of the community is absent. The relationship between sampling and completeness is often graphically shown using a rarefaction curve,^47,54^ which plots diversity of the community as a function of random samples from all samples. Diversity increases with the number of samples collected, until a point of saturation is reached where more sampling does not meaningfully increase diversity, as one has sampled almost all members of the community. Using Chao1 richness, which is a robust richness estimator for microbial count data,^47,48^ we examined the sampling depth of each body site (Figure 1). The rarefaction curve saturates for each body site, indicating that our sampling scheme has captured a relatively complete fusobacterial community. For each patient separately, the overall richness of sequences (alpha diversity) was not significantly different across sampling sites (Kruskal–Wallis rank sum test, *p* = .0625 on Chao1 richness), and for each sampling site, the difference was not significant between different individuals (Kruskal–Wallis rank sum test, *p* = .7673 on Chao1 richness) (Supplementary Figure 3). However, when all sequences at each body site were pooled from all patients, there were significant differences in the overall sequence richness (alpha diversity) by sampling sites. The predicted Chao1 richness from saliva is seen to be significantly greater than that from gastric or colon/pouch, as it lies above the standard error of the latter (*p* < 0.05, Figure 1), indicating a much richer meta-community of unique nodes/strains.

### Phylogenetic placement of Fusobacterium 16S rRNA sequences

**Figure 2.**
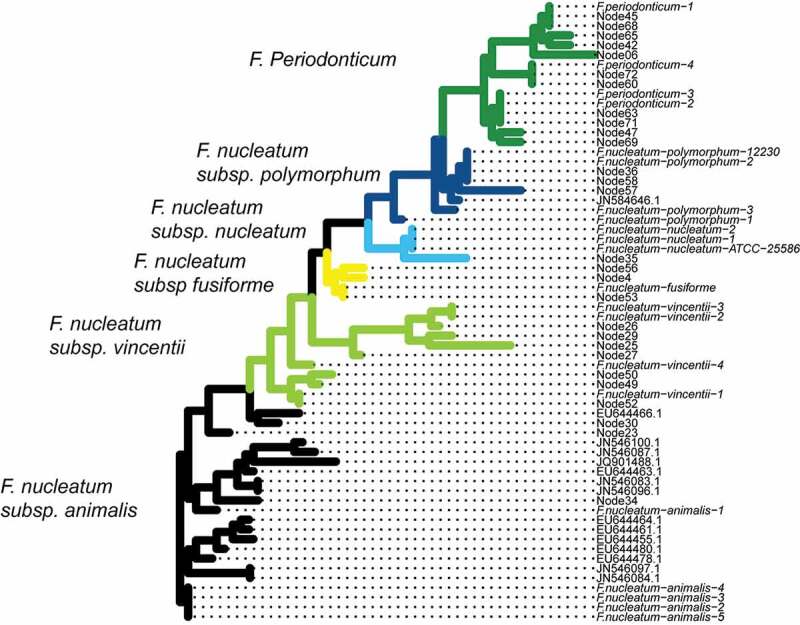
Phylogenetic tree combining the nodes discerned by Minimum Entropy Decomposition (MED) with reference sequences obtained from BLAST. This approach is used to determine the identity of the unknown sequences. Almost all nodes fall into monophyletic clades based on either species or subspecies, which makes phylogeny determination straightforward. The tree is colored by the inferred clades, with names shown to the left of the colored clade

Assigning taxonomy to the 28 nodes/strains will allow us to determine if extra-oral translocation of *Fusobacterium* is particular to the subspecies and/or strain levels. Most 16S rRNA-based classification methods group at the species level. Therefore, we determined taxonomy by creating a phylogenetic tree that combined existing fusobacterial sequences from NCBI and the MED nodes (Figure 2). Different species and subspecies appear to cluster monophyletically, which indicates that fusobacterial subspecies classification is consistent with 16S rRNA sequence diversity. Further, due to the monophyletic groups, we are confident in the assignment of nodes to putative clades, as there appears to be little mixing between different subspecies in the tree structure that would make classification difficult. This allows us to assess the nodes and their translocation ability in the context of fusobacterial phylogeny, to know which species and subspecies each strain/node belongs.

### Fusobacterium community compositions vary according to body sites

**Figure 3.**
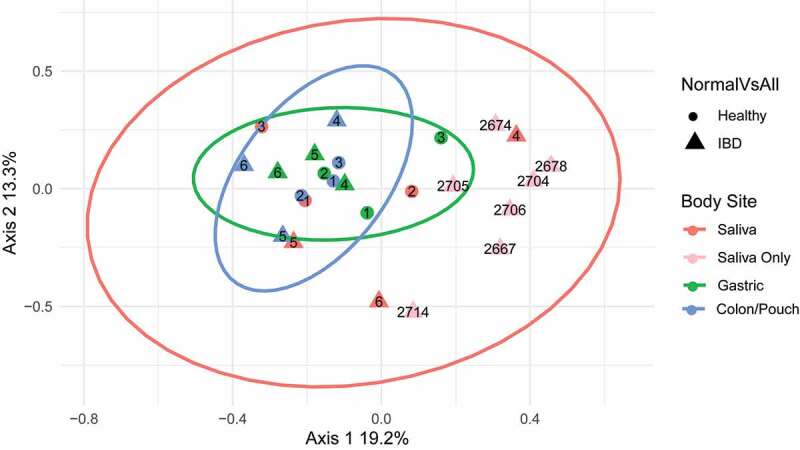
Principle coordinates analysis (PCoA) of *Fusobacterium* communities of all samples. Each symbol represents the community of one body site from one individual, and the distance between them is based upon the Bray-Curtis dissimilarity. The ellipses drawn around the points represent 95% confidence intervals assuming a multivariate t-distribution for each body site using the six matching sample triads. 1–3, healthy controls; 4–6, IBD patients (see Table 1 for subject description). The light pink dots are saliva samples from 7 additional IBD patients without matching gastric or colon/pouch aspirates, all of which fall into the pink ellipse, confirming the predicted variability. Based on the variability of these samples, fusobacterial communities are more closely related in the gastric and colon/pouch than in saliva. Using the matching triads (saliva, gastric aspirate and colon/pouch aspirate) from 6 individuals, we found that different body sites harbor significantly different communities (ANOSIM R: 0.3652, *p* = .003)

Fusobacterial communities from different individuals and at different body sites were compared. Using complete triads (matching saliva, gastric aspirate, colon/pouch aspirate samples) from 3 IBD patients and 3 healthy controls, we found significant compositional differences (beta diversity) at different body sites (ANOSIM R: 0.3652, *p* = .003). However, this difference is not related to disease status when comparing IBD patients to healthy controls (ANOSIM R: −0.08182, *p* = .8512). Furthermore, there was no difference between patients (ANOSIM R = −0.0733, *p* = .86). Overall compositions appeared to vary most between body sites, as seen in Figure 3. By grouping together body sites from the six triads, we generated confidence ellipses representing 95% confidence intervals to demonstrate overall sample variability. Gastric and colon/pouch samples cluster together, while saliva samples have much wider variation. For validation, the seven additional saliva samples fell within the salivary ellipse, regardless of the disease status, i.e. active IBD or IBD in remission (Figure 3 and Table 1). Interestingly, when the saliva samples from 3 healthy subjects were compared with those from the 10 IBD patients, difference between the two groups was detected (ANOSIM R=0.2549, *p* = .0412).

**Figure 4.**
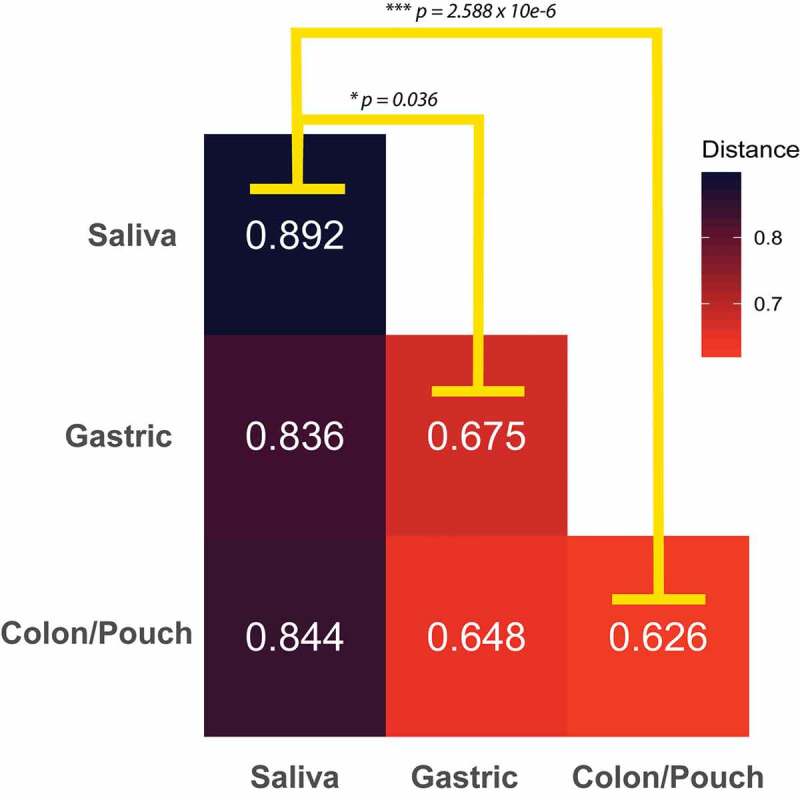
The average Bray-Curtis dissimilarity of *Fusobacterium* communities between body sites. The median distance between body sites from different individuals is calculated using matched saliva, gastric aspirate, and colon/pouch aspirate samples. For example, the upper-left box is the average dissimilarity of a saliva sample to other saliva samples. The oral *Fusobacterium* communities are significantly more dissimilar from each other than from the gastric (Mann-Whitney U-Test, *p* = 0.036) or colon/pouch (Whitney U-Test *p* = 2.588 10^−6^) communities, indicating that there is significantly more variation between oral samples than the GI samples

The high variability of the salivary composition is further demonstrated when comparing the average difference of sample types. The median Bray–Curtis distance between any two saliva samples is significantly larger than the distance between any two gastric or colon/pouch samples (Figure 4). Furthermore, saliva samples exhibit an increased trend of dissimilarity from other sampling sites, while gastric and colon/pouch samples are of similar distance to each other. The reduced composition diversity of *Fusobacterium* in gastric and colon/pouch compared to saliva may indicate the selective pressure during translocation in the GI tract.

**Figure 5.**
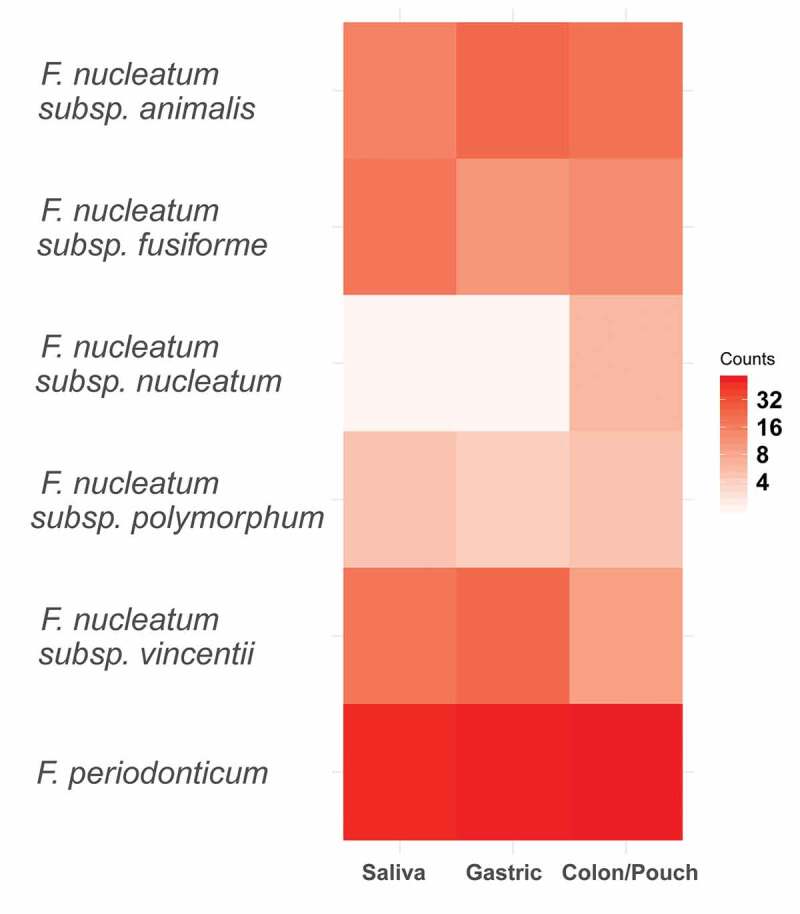
Heatmap of *Fusobacterium* grouped by body site at the clade level as determined by phylogenetic tree. This corresponds to the subspecies level for *F. nucleatum* and the species level for *F. periodonticum. F. periodonticum* is the most abundant clade in each body site. No clades were significantly enriched by the binomial test

**Figure 6.**
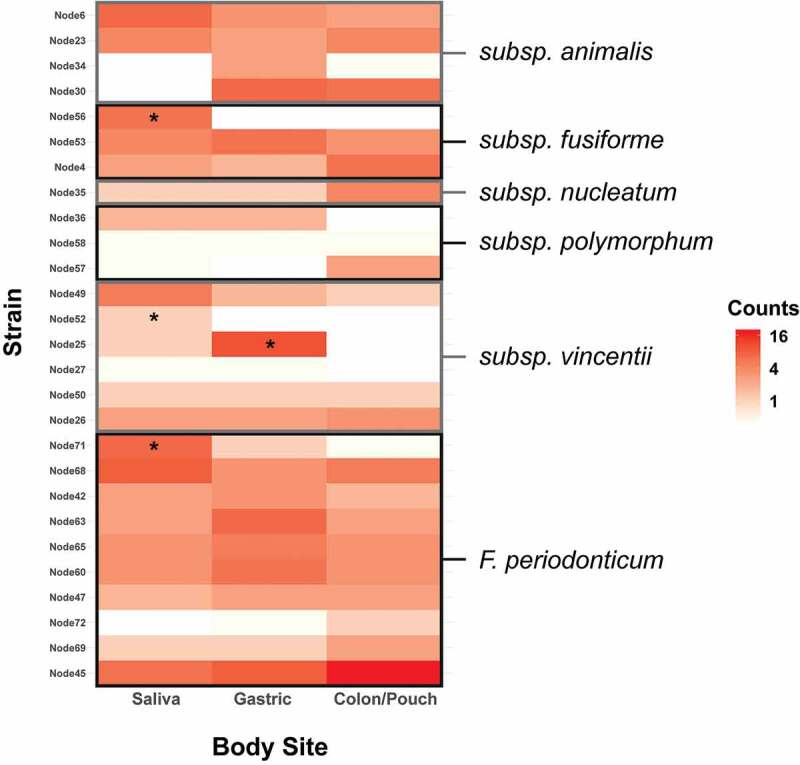
Heatmap of the abundance of distinct *Fusobacterium* nodes at each body site, grouped by subspecies. Enrichment was tested using the binomial test with a Benjamini-Hochberg correction, and significance is indicated by a star. Individual nodes have differing abundance patterns across body sites. *F. nucleatum subsp animalis* tends to persist through colon, while *subsp vincentii* is least likely to colonize the colon. Heatmaps by patients and body sites are shown in Supplementary Figure 4

When each body site was assessed by subspecies, no significant enrichment of any subspecies was observed at any site (Figure 5). However, individual nodes/strains show preponderance in certain body sites. When comparing the variations at the node/strain level, a few exhibited significant differences between body sites (Figure 6). Heatmaps of strains/nodes at the patient and site levels, respectively, are shown in Supplementary Figure 4. The saliva samples had most of the nodes seen across all body sites, while colon/pouch had the fewest. Among these, nodes 30, 4, 35, 57, 26, 72, 69, and 45 were enriched in the colon/pouch compared to saliva, although the difference is not statistically significant, possibly due to limited sample size (Figure 6). In contrast, nodes 56, 36, 49, 52, and 71 were diminished or reduced when translocating from saliva to the GI locations. Node 25 was significantly enriched in gastric but diminished in the lower GI site. At the subspecies level, it appears that *subsp animalis* tends to persist through colon/pouch, while *subsp vincentii* was defective in colonizing the lower GI tract. *F. periodonticum* seems to persist through the GI tract. These results indicate that migration of *Fusobacterium* in the GI tract is a selective process, which occurs predominantly at the strain level, rather than at the subspecies level.

## Discussion

Although oral-fecal transmission has been well documented,^55^ this is the first study to systemically compare fusobacterial diversity in the oral and GI system. We chose saliva as an oral sample not only because it is easy to obtain, but also because the salivary microbial community is more stable than that of dental plaque.^56^ Given the heterogeneity of *F. nucleatum* at the species and subspecies levels, we used MED to analyze 16S rRNA genes to achieve resolution beyond the subspecies level. *Fusobacterium* 16S rRNA genes were amplified by PCR, followed by cloning and Sanger sequencing. The method employed in our study is labor intensive, but it generates highly accurate long reads, allowing for the needed resolution. The primers used in this study, which amplify all five recognized subspecies of *F. nucleatum*, as well as *F. periodonticum*, can be used in future studies in combination with high-throughput long-read sequencing technology, e.g. Pacific Biosciences (PacBio) SMRT sequencing, which would allow for a large number of reads per sample to be generated in a high-throughput manner. Nevertheless, with 381 paired-end reads in the current study, we have shown that *Fusobacterium* differ between body sites. Our study also reveals the phylogenetic relationship between *F. nucleatum* subsepcies.

Using matched oral, gastric and colon/pouch samples from IBD patients and healthy controls, along with saliva samples from additional IBD patients, we have observed emerging patterns. *Fusobacterium* community compositions in saliva are the most diverse, significantly more so than in the GI locations, and may be related to the disease status. Diversity in the GI sites is signficantly reduced, possibly due to intrinsic selective pressure affecting *Fusobacterium* colonization, e.g. acid in the stomach, digestive enzymes in the small intestine, and colonization resistance in the colon. Only those that are capable to overcome such environmental obstacles can colonize the GI niches. The similarity between the gastric and colon/pouch communities was unlikely due to polyethylene glycol-based colonic preparation because it would have altered the oral community as well. Instead, it suggests that gastric acid may be a major limiting factor for fusobacterial translocation down the GI tract. This is consistent with previous report that gastric acid affects the infectious potential of ingested bacterial pathogens.^57^

Although *subsp animalis* tends to persist though the GI tract, and *subsp vincentii* appears to be least likely to colonize the colon/pouch, no clades are significantly enriched by the binomial test. Instead, the adaptation of *Fusobacterium* to gastro intestine appears to be at the strain/node level, rather than at the species or subspecies levels. This is consistent with a previous report that most subspecies of *Fusobacterium* are detected in CRC.^28^ Therefore, subspecies classification alone is not sufficient to identify the translocation and disease potentials of individual *Fusobacterium* strains. Future studies will investigate specific virulence factors associated with the transmissible strains/nodes to understand how the translocation occurs.

## Supplementary Material

Supplemental Material

## Data Availability

Genbank submission reference is SUB7794064. Accession numbers for each individual sequence are MT780937-MT781319.
