# Supplementary material for: Analysis of 16S rRNA genes reveals reduced Fusobacterial community diversity when translocating from saliva to GI sites
Source: Gut Microbes. 2020 Oct 15;12(1):1814120. doi: 10.1080/19490976.2020.1814120 (PMC7577115; doi:10.1080/19490976.2020.1814120)
Supplement: Supplemental Material [file KGMI_A_1814120_SM1728.zip › Supplementary information/Supplementary Table 1.docx]

**Supplementary Table 1.**

| **Participant No.** | **Site** | **No. sequences** | **No. aligned sequences after QC and MED** |
| --- | --- | --- | --- |
| 1 | Saliva | 21 | 18 |
|  | Gastric | 20 | 18 |
|  | Colon/Pouch | 22 | 21 |
| 2 | Saliva | 20 | 17 |
|  | Gastric | 20 | 16 |
|  | Colon/Pouch | 22 | 19 |
| 3 | Saliva | 20 | 20 |
|  | Gastric | 20 | 20 |
|  | Colon/Pouch | 20 | 8 |
| 4 | Saliva | 20 | 17 |
|  | Gastric | 21 | 17 |
|  | Colon/Pouch | 21 | 17 |
| 5 | Saliva | 21 | 20 |
|  | Gastric | 21 | 21 |
|  | Colon/Pouch | 22 | 20 |
| 6 | Saliva | 21 | 16 |
|  | Gastric | 22 | 22 |
|  | Colon/Pouch | 22 | 21 |
| 2667 | Saliva | 20 | 13 |
| 2674 | Saliva | 29 | 10 |
| 2678 | Saliva | 18 | 8 |
| 2704 | Saliva | 21 | 8 |
| 2705 | Saliva | 19 | 5 |
| 2706 | Saliva | 20 | 7 |
| 2714 | Saliva | 19 | 2 |
| **Total** | | **522** | **381** |
