# Supplementary figures and images for: Analysis of 16S rRNA genes reveals reduced Fusobacterial community diversity when translocating from saliva to GI sites
Source: Gut Microbes. 2020 Oct 15;12(1):1814120. doi: 10.1080/19490976.2020.1814120 (PMC7577115; doi:10.1080/19490976.2020.1814120)

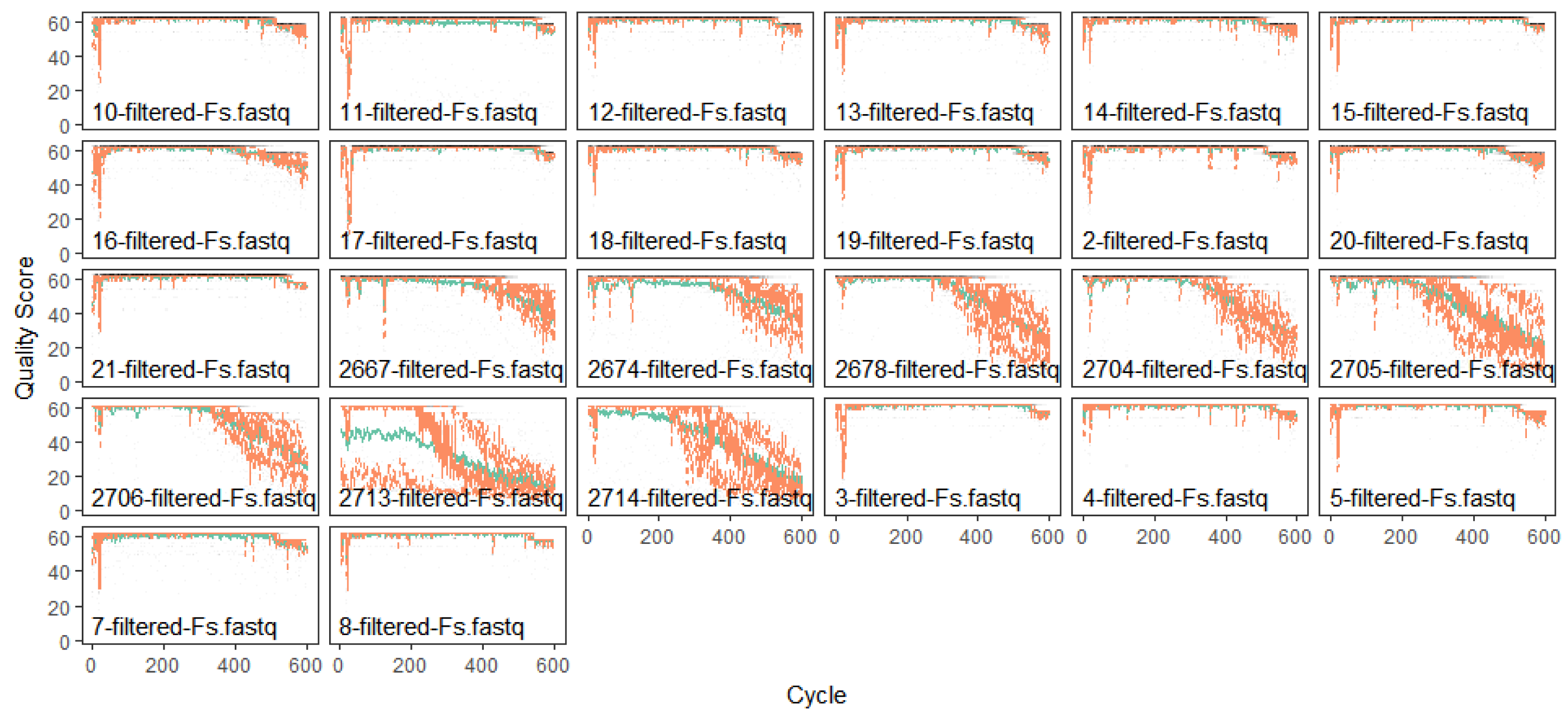

Supplement: Supplemental Material [file KGMI_A_1814120_SM1728.zip › Supplementary information/Supplementary Figure 1A.tif]

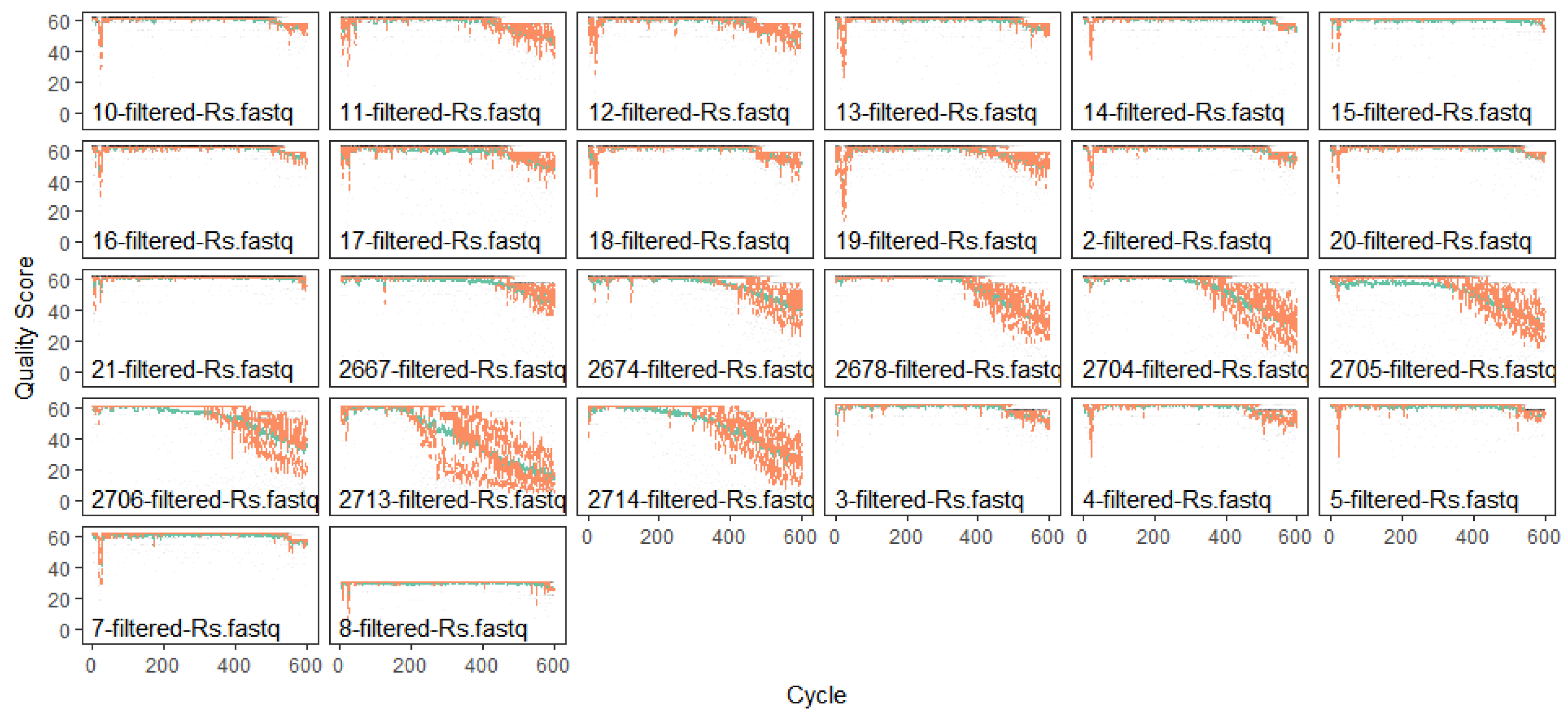

Supplement: Supplemental Material [file KGMI_A_1814120_SM1728.zip › Supplementary information/Supplementary Figure 1B.tif]

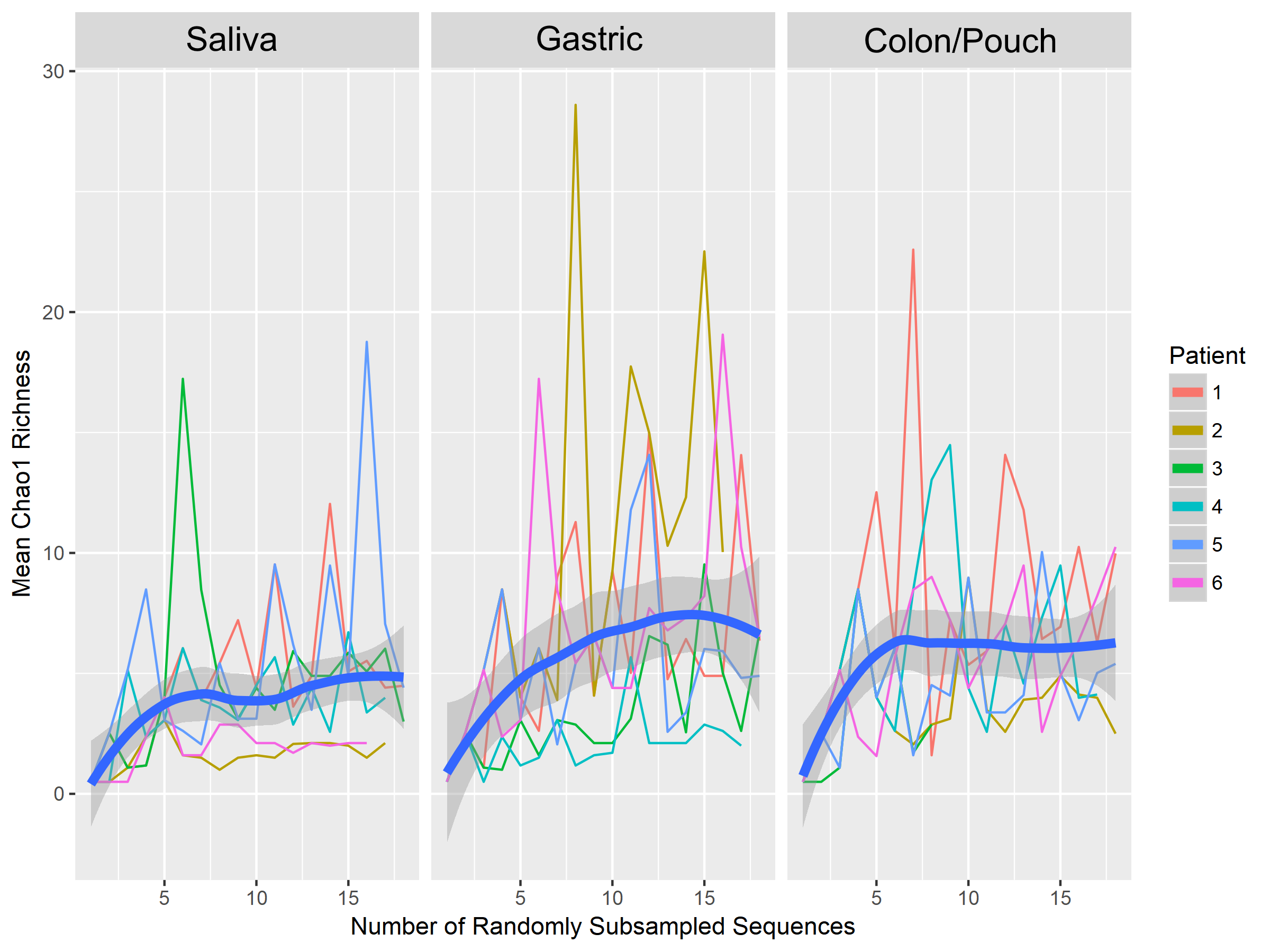

Supplement: Supplemental Material [file KGMI_A_1814120_SM1728.zip › Supplementary information/Supplementary Figure 3.tif]

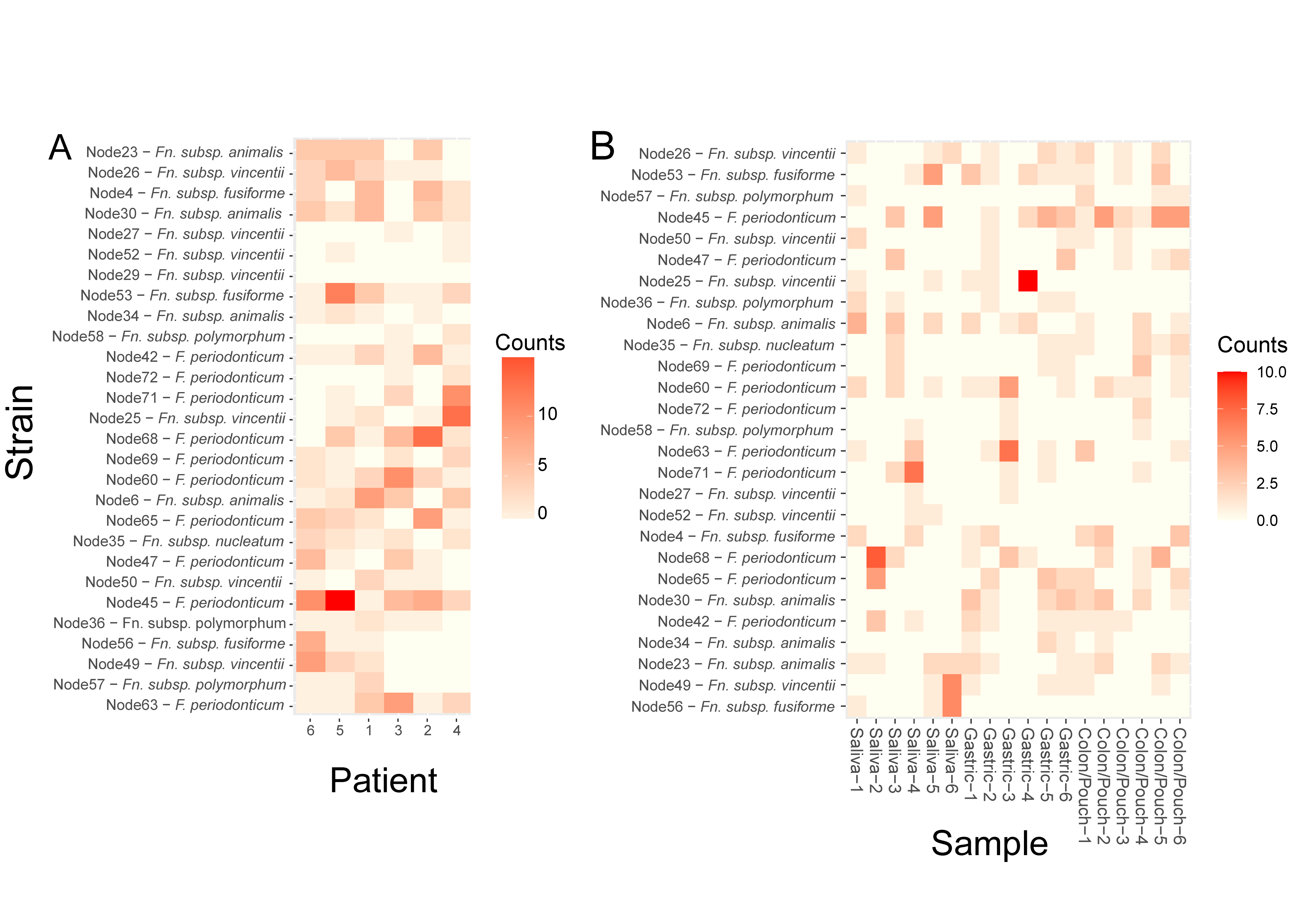

Supplement: Supplemental Material [file KGMI_A_1814120_SM1728.zip › Supplementary information/Supplementary Figure 4.tiff]
